# Can local staff reliably assess their own programs? A confirmatory test-retest study of Lot Quality Assurance Sampling data collectors in Uganda

**DOI:** 10.1186/s12913-016-1655-4

**Published:** 2016-08-17

**Authors:** Colin A. Beckworth, Robert Anguyo, Francis Cranmer Kyakulaga, Stephen K. Lwanga, Joseph J. Valadez

**Affiliations:** 1 Liverpool School of Tropical Medicine, Pembroke Place, Liverpool, L3 5QA United Kingdom; 2 Uganda Martyrs University, Nkozi, Uganda; 3 Uganda Christian University, Mukono, Uganda; 4 Management Sciences for Health, USAID STAR-E project, Mukono, Uganda

**Keywords:** LQAS, Lot Quality Assurance Sampling, Test retest, Cohen’s kappa, Bias

## Abstract

**Background:**

Data collection techniques that routinely provide health system information at the local level are in demand and needed. LQAS is intended for use by local health teams to collect data at the district and sub-district levels. Our question is whether local health staff produce biased results as they are responsible for implementing the programs they also assess.

**Methods:**

This test-retest study replicates on a larger scale an earlier LQAS reliability assessment in Uganda. We conducted in two districts an LQAS survey using 15 local health staff as data collectors. A week later, the data collectors swapped districts, where they acted as disinterested non-local data collectors, repeating the LQAS survey with the same respondents. We analysed the resulting two data sets for agreement using Cohens’ Kappa.

**Results:**

The average Kappa score for the knowledge indicators was k = 0.43 (SD = 0.16) and for practice indicators k = 0.63 (SD = 0.17). These scores show moderate agreement for knowledge indicators and substantial agreement for practice indicators. Analyses confirm that respondents were more knowledgeable on retest; no evidence of bias was found for practice indicators.

**Conclusion:**

The findings of this study are remarkably similar to those produced in the first reliability study. There is no evidence that using local healthcare staff to collect LQAS data biases data collection in an LQAS study. The bias observed in the knowledge indicators was most likely due to a ‘practice effect’, whereby respondents increased their knowledge as a result of completing the first survey; no corresponding effect was seen in the practice indicators.

**Electronic supplementary material:**

The online version of this article (doi:10.1186/s12913-016-1655-4) contains supplementary material, which is available to authorized users.

## Background

Health surveys are, arguably, the “the primary method for estimating population-level intervention coverage in low- and middle-income countries” [1]. Despite progress made since the World Health Organisation’s (WHO) Advisory Panel on Health Statistics called for more and better health statistics [2], there are still challenges to overcome. Routine health management information systems (HMIS) can provide valuable health service demand-side information, but being a convenience sample is inadequate for measuring coverage and supporting related programmatic decision-making [3]. Whilst macro-level surveys provide detailed high quality information, they do not provide the local-level information that is necessary for local program management. More research about data collection techniques which can routinely provide information at the local level is in demand and needed [1]. Lot Quality Assurance Sampling (LQAS) may contribute to satisfying this need [4].

LQAS is a classification method derived from the original work of Dodge and Romig [5], which together with that of Shewhart [6], grew to become Statistical Quality Control. During the 1980’s it made its transition into the health sciences, gaining wide appeal [7]. During the 1990s WHO favourably reviewed the methodology as providing regular coverage data at the local level [8].

LQAS has two stages, but first requires dividing a program area (such as a district) into smaller sub-areas (or sub-districts) called Supervision Areas (SA). In the first stage a random sample is collected within each SA and used to classify the SA as acceptably or unacceptably performing according to a predetermined threshold [9]. In the second stage the data from SA are aggregated to measure the prevalence of program areas as a whole. This methodology has been extensively used by UN agencies, Ministries of Health and NGOs to periodically collect data to manage health programs using local health staff to collect data [8].

However, as LQAS is intended for use by local program managers, the question must be examined as to whether local health staff produce biased results as they are responsible for implementing the programs they also assess. This question is not trivial as bias is described as “the greatest threat to reliability and validity” of collected data [10].

An initial, albeit small scale, study assessing whether local data collectors are a source of bias in LQAS survey [11], found no evidence to support the hypothesis that they bias the data they collect. However, that study was restricted to one district, and the second set of dis-interested data collectors came from the same district; also the sample size was small consisting of 76 participants. This current study is designed as a larger confirmatory test-retest study to measure inter-observer reliability of LQAS data collection. The study was located in two districts in Uganda.

## Methods

We used a test-retest methodology to compare the inter-observer reliability between two groups of data collectors when carrying out an LQAS survey. Inter-observer reliability is the degree of agreement between two different data collectors when making observations of the same phenomenon [12]. Test-retest measures the inter-observer reliability of the data collected by two independent sets of data collectors [13]. Provided the phenomenon under examination has not changed, the two sets of observations should be the same; the greater the agreement between the two observations, the greater the inter-observer reliability.

In our study, the first group of data collectors was an ‘interested’ group responsible for managing the service provision being assessed. The second group was a ‘disinterested’ group who were not responsible for service provision and/or management in the same area. We introduced no other change. We used this test–retest study to examine the agreement of the information provided by data collectors with a vested interest in the results (the interested data collectors) as opposed to those without a vested interest (the disinterested data collectors) and whether the former collect biased data.

The study site was two districts in Uganda 200 km apart, Buikwe and Bukomansimbi. These two districts had previously carried out several rounds of LQAS using 15 data collectors in each district. Each district was subdivided into five SAs. For the ‘test’ phase of the research, the data collection teams administered a questionnaire in their home districts using the standard method [14]. Since the teams were in their home districts where they were responsible for providing services, we labelled them as ‘interested’ data collectors. One week later, the 15 data collectors from Bukomansimbi moved to Buikwe, and the 15 data collectors from Buikwe moved to Bukomansimbi. The teams then carried out the ‘Retest’, using the same questionnaire with the same respondents as previously surveyed. However, since the teams were no longer in their home districts and had no responsibility for service provision, we labelled them as ‘disinterested’ data collectors. Nineteen respondents were selected randomly from each SA for the LQAS classification. With *n =* 19 alpha and beta errors do not exceed 0.10 for high or very low performing SA [14]. The total district sample is *n =* 95 (5 × 19). Therefore, *n =* 190 respondents for the full study. We employed probability proportional to size sampling to select 19 interview locations in each SA and segmentation sampling to select respondent households. The respondents were confirmed as being the same respondent by checking their name; village; whether they had given information for a survey a week previously and where possible by their mobile phone number.

The data collection teams were selected by the senior district health managers, who were all experienced with using LQAS data. We requested the district health managers to select the data collectors who had collected data during previous rounds of LQAS; the teams comprised 21 clinical staff and nine non-clinical support staff. Twenty-five of the staff were full time employees of the districts; the other five were periodically employed by the health district when needed. All of the data collectors attended a four-day LQAS data collector training course from 9th to the 13th of September 2013. The data collectors were not informed of the true aim of the study so as not to prejudice the data collection. Rather they were told that the study was being carried out to examine operational issues associated with implementing LQAS in the districts. After the completion of the study, the teams were informed of the true reason for the study and results were fed back to the districts—which is the intention of LQAS assessments.

The questionnaire was adapted from a pre-tested LQAS questionnaire for mothers of children 0–11 months old used previously in multiple districts throughout Uganda to explore knowledge and practices around malaria, TB, HIV and other sexually transmitted infections (STI). The questionnaire was adapted so that questions for which the answer could change between the test and the retest were excluded. The questionnaire was the same one as used in a previous smaller LQAS reliability study [11]. Therefore, the results for this study are directly comparable to the previous LQAS reliability study. The resulting questionnaire produced 23 indicators pertaining to the respondents’ knowledge and 14 indicators pertaining to practice. The data were double entered using EpiInfo 7 and analysed using SPSS v21.

The test and retest data were analysed for agreement using Cohen’s Kappa. This test measures agreement between two scores and is widely used in test-retest studies [15]. We chose Cohen’s Kappa because since it is an appropriate statistic to measure inter-rater reliability with nominal data [16], and other authors have used Kappa for this type of analysis [17, 18]. The Kappa score ranges between 0 (no agreement) and 1 (complete agreement) [19], the interpretation for which we include in Table 1. However, we noted that because of the base rate problem, Kappa can be unstable at very high or very low prevalence [20]. We therefore excluded from our analysis any indicator where the “a” or “d” cells include in the cross tabulation were <5.

**Table 1:** Standard Categories to Interpret Kappa values (Landis & Koch 1977)

Kappa Value (k)	Strength of Agreement
<0.00	Poor
0.00–0.20	Slight
0.21–0.40	Fair
0.41–0.60	Moderate
0.61–0.80	Substantial
0.81–1.00	Almost Perfect

Ethical approval for this research was granted by Makerere School of Public Health, and approval was given by the Uganda National Council of Science and Technology. Written informed consent was obtained for all participants in the study, and the consent form was approved by the ethics committee.

## Results

Table 2 shows the coverage estimates for the practice indicators on the test and retest for the two districts. Table 3 does the same for knowledge indicators. The results from the test and the retest were then analysed using a paired *t*-test; the resulting *p* values are displayed in the column following the test and retest coverage estimates. The *p* values range from <0.001 to 1 for knowledge and 0.083 to 1 for the practice indicators. Of the 34 results analysed for the knowledge indicators, only six had a *p* value of ≤0.05. Of these six, only one was higher on the test, when the interested data collectors were collecting the data. None of the 26 results analysed for the practice indicators had a *p* value ≤0.05.

**Table 2:** Coverage Estimates for Practice Indicators

		Bukomansimbi			Buikwe		
		Test	Retest	*p*	Test	Retest	*p*
1	Could show an ANC card	58.9 %	62.1 %	0.32	73.7 %	69.5 %	0.103
2	Gave birth in health facility	74.7 %	76.8 %	0.158	74.7 %	80.0 %	0.132
3	Gave birth with a skilled birth attendant	71.6 %	69.5 %	0.566	73.7 %	77.9 %	0.25
4	Has ever used a condom	57.9 %	53.7 %	0.287	71.6 %	71.6 %	1
5	Owns a LLIN	85.3 %	84.2 %	0.765	82.1 %	76.8 %	0.096
6	Received IPT whilst pregnant	46.3 %	54.7 %	0.45	58.9 %	54.7 %	0.348
7	Received PMTCT counselling	76.8 %	77.9 %	0.798	83.2 %	87.4 %	0.25
8	Received result	81.1 %	80.0 %	0.741	85.3 %	86.3 %	0.765
9	Slept under a mosquito net whilst pregnant	83.2 %	89.5 %	0.083	82.1 %	83.2 %	0.708
10	Slept under mosquito net every night whilst pregnant	69.5 %	72.6 %	0.551	77.9 %	75.8 %	0.566
11	Took an HIV test	82.1 %	83.2 %	0.741	90.5 %	90.5 %	1
12	Went for 4 ANC visits	44.2 %	44.2 %	1	42.1 %	35.8 %	0.083
13	Was counselled to take an HIV test	86.3 %	85.3 %	0.783	93.7 %	92.6 %	0.708

**Table 3:** Coverage Estimates for Knowledge Indicators

		Bukomansimbi			Buikwe		
		Test	Retest	*p*	Test	Retest	*p*
1	Knows at least one way that the risk of mother to child transmission can be reduced	88.4 %	89.5 %	0.708	90.5 %	93.7 %	0.32
2	Knows at least two actions to take if they have an STI	33.7 %	41.1 %	0.288	34.7 %	37.9 %	0.615
3	Knows at least two signs of STI in females	77.9 %	82.1 %	0.158	81.1 %	91.6 %	0.001
4	Knows at least two signs of STI in males	45.3 %	54.7 %	0.038	58.9 %	65.3 %	0.158
5	Knows of at least one STI other than HIV	86.3 %	89.5 %	0.259	94.7 %	96.8 %	0.158
6	Knows that HIV can be transmitted to an infant during breastfeeding	57.9 %	62.1 %	0.397	65.3 %	70.5 %	0.3
7	Knows that HIV can be transmitted to an infant during delivery	73.7 %	68.4 %	0.253	69.5 %	74.7 %	0.32
8	Knows that HIV can be transmitted to an infant during pregnancy	24.2 %	28.4 %	0.453	36.8 %	18.9 %	0.002
9	Knows that the risk of mother to child transmission can be reduced	90.5 %	91.6 %	0.657	91.6 %	95.8 %	0.208
10	Knows the three strategies to prevent HIV infection	5.3 %	7.4 %	0.482	7.4 %	12.6 %	0.167
11	Knows where to get treatment for STI	77.9 %	85.3 %	0.07	86.3 %	95.8 %	0.006
12	Knows whether HIV is transmitted via Mosquitoes	63.2 %	68.4 %	0.198	61.1 %	63.2 %	0.62
13	Knows whether HIV is transmitted via sharing food with infected person	82.1 %	88.4 %	0.109	76.8 %	84.2 %	0.109
14	Knows whether HIV is transmitted via sharing toilets	81.1 %	81.1 %	1	74.7 %	83.2 %	0.02
15	Knows whether HIV is transmitted via sharing utensils	67.4 %	88.4 %	0	77.9 %	74.7 %	0.494
16	Knows whether HIV is transmitted via touching infected person	89.5 %	91.6 %	0.566	85.3 %	91.6 %	0.096
17	Knows whether HIV is transmitted via witchcraft	85.3 %	86.3 %	0.62	88.4 %	91.6 %	0.47

Tables 4 and 5 show the Kappa scores for the knowledge and practices indicators respectively. The average Kappa score for the knowledge indicators was k = 0.43 (SD = 0.16) and for practice indicators k = 0.63 (SD = 0.17), (Tables 4&5). These scores show moderate agreement for knowledge indicators and substantial agreement for practice indicators.

**Table 4:** Kappa Scores for Knowledge Indicators

No.	Indicator	Interviewer Agreement		Disagreement		Kappa	Strength of Agreement
		yes	no	yes & no	no & yes		
1	Know of at least one STI other than HIV	11	170	7	2	0.69	Substantial
2	Know whether HIV is transmitted via Mosquitoes	53	106	19	12	0.65	Substantial
3	Know whether HIV is transmitted via sharing toilets	27	141	15	7	0.64	Substantial
4	Know at least two signs of STI in females	37	116	29	8	0.54	Moderate
5	Know at least two signs of STI in males	105	45	28	12	0.54	Moderate
6	Know at least one way that the risk of mother to child transmission can be reduced	10	164	10	6	0.51	Moderate
7	Know that HIV can be transmitted to an infant during breastfeeding	46	99	27	18	0.49	Moderate
8	Knows the risk of mother to child transmission can be reduced	7	168	10	5	0.44	Moderate
9	Knows HIV can be transmitted to an infant during delivery	32	114	22	22	0.43	Moderate
10	Know where to get treatment for STI	13	151	21	5	0.43	Moderate
11	Know whether HIV is transmitted via sharing utensils	23	127	28	11	0.42	Moderate
12	Know whether HIV is transmitted via touching infected person	9	159	14	7	0.4	Moderate
13	Know whether HIV is transmitted via sharing food with infected person	16	141	23	10	0.39	Fair
14	Know the three strategies to prevent HIV infection	164	5	14	7	0.27	Fair
15	Know that HIV can be transmitted to an infant during pregnancy	109	22	23	36	0.22	Fair
16	Know whether HIV is transmitted via witchcraft	6	150	19	14	0.17	Slight
17	Know at least two actions to take if they have an STI	81	31	44	34	0.12	Slight
	Mean Values					0.43

**Table 5:** Kappa Scores for Practice Indicators

No.	Indicator	Interviewer Agreement		Disagreement		Kappa	Strength of Agreement
		yes	no	yes & no	no & yes		
1	Went for 4 ANC visits	137	41	3	9	0.86	Almost Perfect
2	Could show an ANC card	57	118	7	8	0.82	Almost Perfect
3	Gave birth with a skilled birth attendant	36	142	9	3	0.82	Almost Perfect
4	Gave birth in health facility	38	139	10	3	0.81	Almost Perfect
5	Have ever used a condom	59	111	8	12	0.77	Substantial
6	Received result	22	148	10	10	0.62	Substantial
7	Slept under a mosquito net whilst pregnant	20	151	13	6	0.62	Substantial
8	Took an HIV test	16	155	10	9	0.57	Moderate
9	Received PMTCT counselling	22	141	16	11	0.53	Moderate
10	Slept under mosquito net every night whilst pregnant	31	122	19	18	0.5	Moderate
11	Received IPT whilst pregnant	129	25	16	20	0.46	Moderate
12	Were counselled to take an HIV test	10	160	9	11	0.44	Moderate
13	Own a LLIN	24	125	17	24	0.4	Moderate
	Mean Values					0.63	

Further analyses explored the direction of the discordant results to assess bias in health worker interviews. A respondent who answers correctly to a knowledge question (such as knowing the ways HIV can be transmitted to an infant) or who responds that they practice a desirable health behaviour (such as a mother going for four or more antenatal care visits) is scored as giving a ‘positive’ response. Positive responses show that health services are performing well in a particular area. Bias can be defined as systematic error, as compared with random error [21]. Our survey examined knowledge and practices of Ugandans concerning malaria, TB and HIV/STI. If local health workers collected biased data then their responses should be consistently and significantly more positive than those of the disinterested data collectors.

We categorized the indicators as either knowledge or practice. Knowledge indicators measured whether a respondent could correctly state key health messages; practice indicators measured whether respondents had practiced key health behaviours. We separated the indicators so that we could examine the results for bias by indicator type.

**Fig. 1:**
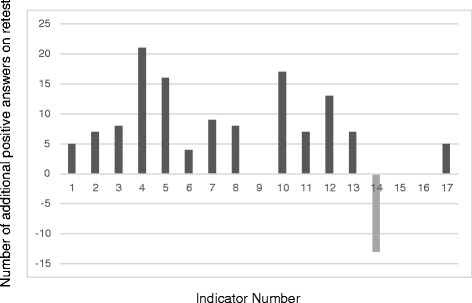
Additional Positive Answers on the Retest for Knowledge Indicators

**Fig. 2:**
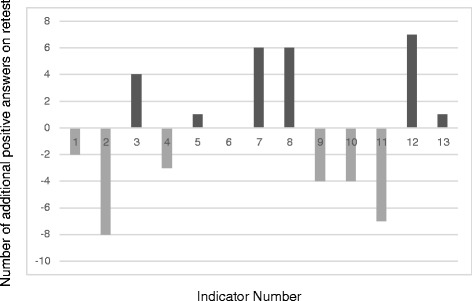
Additional Positive Answers on the Retest for Practice Indicators

On average, the additional number of positive responses on the retest was 6.7 for knowledge indicators (95 % CI =3.0 to 10.4) and −0.2 for the practice ones (95 % CI = −2.9 to 2.5) (Figs. 1 and 2). These results indicate that respondents were significantly more knowledgeable during the retest with the disinterested data collectors; 13 of the 17 knowledge indicators show positive values during the retest (Fig. 1) with only one negative value. The practice indicators show no difference between the test and retest (Fig. 2). Six values are positive (above the x axis) and six negative (below the x axis). These data reveal no significant or consistent directional difference for the practice indicators and therefore, no bias. Data for the test, retest, and the questionnaire used are freely available as supplementary materials (Additional files 1, 2 and 3).

## Discussion

Our study found substantial agreement for practice indicators and moderate agreement for knowledge indicators. We found evidence of bias for the knowledge indicators but not the practice indicators, as the respondents were more knowledgeable on the retest when interviewed by non-interested data collectors. These findings are strikingly similar to the first LQAS reliability study carried out in 2012 [11].

The average Kappa score for knowledge indicators was k = 0.43 in both the first and this current study (SD = 0.13 and SD = 0.16, respectively). There were on average 5.9 (95 % CI: 4.2 to 7.6) more positive answers on the retest for study one, and 6.7 (95 % CI = 3.0 to 10.4) for the current one. These results support the test-hypothesis that local managers do not collected biased data indicating favourable performance.

For practice indicators the average Kappa score was k = 0.73 (SD = 0.16) for the first study,and k= 0.63 (SD = 0.17) for the current one. Both Kappa scores indicate ‘substantial’ agreement between the two data collection teams [19]. There were on average −0.5 (95 % CI: −2.1 to 1) more positive answers in the first test, and −0.2 (95 % CI: −2.9 to 2.5) more positive answers on the second test. These similarities in the findings indicate that the current study results confirm those of the original one.

The current study’s design has several important improvements compared to the former one. Firstly, the sample size was larger. In the original reliability study *n =* 76 whilst in this study *n =* 190. Secondly, in the first study, the data were collected in one district. Hence, there was possibility of contamination of results by the data collectors, despite the authors’ efforts to ensure that the data collectors held responsibilities only in the area where they carried out the test and had no responsibilities in areas where they carried out the retest. The contamination is possible since two of the 10 data collectors had responsibilities cutting across the test and retest areas and all the data collectors worked for the same district health authority. In the current study, the test and retest areas were two districts over 200 km apart. There was therefore virtually no chance that the data collectors could have responsibility for services in both the test and retest areas.

The original reliability study [11] concluded that the evidence of bias revealed on the retest had three possible explanations. Firstly, using interested data collectors could bias findings by making respondents appear less knowledgeable than they actually were (an unlikely possibility); secondly, using non-interested data collectors could bias findings by making respondents more knowledgeable than they actually were (also unlikely); and thirdly, an increase in knowledge in the re-test could be due to a practice effect, which is bias introduced at the retest stage because the respondent has become familiar with the test, or, in this case, the survey questionnaire [22]. The first reliability study concluded that the most likely explanation for the higher knowledge indicators at the re-test was a practice effect.

Only six out of 50 indicators (25 in each district) showed a difference between the test and retest with a *p* value ≤0.05, and all of these were for knowledge indicators. Of these six knowledge indicators, five showed an increase in knowledge between the test and the retest. Just one indicator out of 50 had respondents more knowledgeable on the test (when interviewed by the interested data collectors) than on the retest with a *p* value of ≤0.05 (knows that HIV can be transmitted to an infant during pregnancy). Therefore, we think the practice effect is the likely explanation for the higher knowledge indicators found in the current study. Although we classified the indicators using the widely accepted categories ranging from *poor* to *almost perfect agreement* given [19], these categories are arbitrary [23]. There are other examples of test-retest research with which we compare our study results. Drum et al. [24] pretested a questionnaire concerning disability access in clinics in North America. Their initial test resulted in a mean Kappa score of 0.61. Whilst they regarded this result as “acceptable”, after repeated revisions to the questionnaire and subsequent re-tests they increased the Kappa score to 0.97. However, the authors gave no indication of the sample size and presented no table with results. Flisher et al. [25], however, gave greater detail about their reliability study of a Mental Health Needs Assessment tool. They found very similar results to our survey, with an average Kappa of 0.63, but they also record considerable variation depending on the indicator (Kappa range: 0.25 to 0.81). They concluded that the tool was “relatively reliable”. However, the authors had the advantage of reviewing similar test retest studies using the same tool in a variety of settings with which they compared their results.

Whereas our study is comparable to these previous studies, the subjects and research designs were considerably different. We could appraise our results in a more in-depth manner if test re-test data were available for other LQAS or major health surveys used internationally. For example, UNICEF’s Multiple Indicator Cluster Surveys, and the Demographic and Health Surveys are large macro surveys of health and demographic variables; yet, there are no reliability studies available for either one. The variability of the Kappa statistics across the indicators in our study suggests that certain types of questions may be more reliable than others. In our current study and in the previous one, the practice indicators appear to be more reliable than knowledge indicators.

Another way to classify the indicators is by the way they are calculated. Some indicators are calculated using simple yes/no questions, while others use more complicated question forms where the data collectors must select multiple responses from a list. The average Kappa score for indicators of the first type is 0.55, whereas for the second type the result is 0.44. This suggests that indicators calculated using select multiple questions are less reliable than the indicators calculated using yes/no questions. Further research should be carried out to assess the relative reliability of various question types.

An important limitation of this study is the lack of test-retest reliability data available for other major health surveys; therefore, it is difficult for us to define an acceptable level of reliability. The original and current studies are at the vanguard of such studies. Also, this confirmatory study was carried out in two districts with very similar characteristics to the initial study (Pallisa). There is still need to carry out a similar study in a considerably different setting for further comparison. On the retest, a practice effect was observed when examining the knowledge variables, but this is an assumption, which requires further study and confirmation. The carryover effect—the respondents may have remembered the answer they gave in the test and repeated that rather than the recalled the actual variable under study - may also have affected the results of the study, even though a week was given between the test and retest.

## Conclusion

The findings of this study are remarkably similar to those produced in the first reliability study. There is no evidence that using local healthcare staff to collect LQAS data biases data collection in an LQAS study. The bias observed in the knowledge indicators was most likely due to a ‘practice effect’, whereby respondents increased their knowledge as a result of completing the first survey, as no corresponding effect was seen in the practice indicators. Local health managers when well trained in survey methods are capable of collecting reliable information they then use for program management. Perhaps their data are reliable because they use the data and therefore care about its quality.

## Additional files

Additional file 1: 0 to 5 months test. (XLSX 156 kb) Additional file 2: 0 to 5 months retest. (XLSX 155 kb) Additional file 3: Questionnaire final Copy. (PDF 517 kb)

## Abbreviations

CI: Confidence interval
HIV: Human immunodeficiency virus
HMIS: Health management information system
LQAS: Lot quality assurance sampling
NGO: Non-governmental organisation
SA: Supervision area
SD: Standard deviation
STI: Sexually transmitted infection
TB: Tuberculosis
UN: United nations
UNICEF: United nations children’s fund
WHO: World health organisation
