# Supplementary material for: Can local staff reliably assess their own programs? A confirmatory test-retest study of Lot Quality Assurance Sampling data collectors in Uganda
Source: BMC Health Serv Res. 2016 Aug 17;16:396. doi: 10.1186/s12913-016-1655-4 (PMC4989458; doi:10.1186/s12913-016-1655-4)
Supplement: Additional file 3: — Questionnaire final Copy. (PDF 517 kb) [file 12913_2016_1655_MOESM3_ESM.pdf]

Lot Quality Assurance Sampling survey in Buikwe and Bukomansimbi Districts, Uganda

QUESTIONNAIRE FOR MOTHERS OF CHILDREN (0 – 11 MONTHS)

IDENTIFICATION	CODES
QUESTIONNAIRE IDENTIFICATION	☐☐☐☐☐
LQAS NUMBER OUT OF 19________________	☐☐
SUPERVISION AREA ________________	☐☐
District________________	
County________________	
Sub-County________________	
Parish________________	
L.C. 1 (Village, Zone)________________	
Household Head________________	
Name of Respondent________________	
Mobile No Respondent:________________	
Interviewer : ________________	
Mobile No Interviewer:________________	
Date of Interview ------ / ------ / ------ Day / Mo / Yr	
Checked by (Supervisor) ________________	
Date checked ------ / ------ / ------ Day / Mo / Yr	

Name of Village Guide:________________ Phone Contact Guide:____________

1. **Fill in the front sheet ONLY in pen. Use pencil for the rest of the questionnaire**
2. **Fill in the front sheet neatly and clearly**
3. **Draw a clear directional map at the back of the Questionnaire**

UGANDA CHRISTIAN UNIVERSITY
A Centre of Excellence in the Heart of Africa

Uganda Martyrs University

LSTM LIVERPOOL SCHOOL OF TROPICAL MEDICINE

INTRODUCTION AND CONSENT

Greetings.

My name is ___________________ and I work with _____________________. We are conducting a survey about the health of people in our communities. We are interested in finding out health facts about mothers of infants, and will be talking to many mothers of infants in the area. This information will help us understand the health needs of your community, and will help us to find better ways to gather information on health of your community. The interview takes about **35 minutes** to complete. Participation is completely voluntary, no payment will be given, and if you don't wish to take part, you will not be penalised in any way.

If you agree to participate, someone will also come back in one week's time to ask more questions on the same subject.

The survey is being organised by Uganda Christian University (UCU), Uganda Martyrs University (UMU) and Liverpool University (LSTM) in the UK, and the supervisors are **Francis Cranmer Kyakulaga** and **Robert Anguyo**, from UCU and UMU respectively.

We very much appreciate your participation in this survey and I would like to ask you some questions concerning several health issues, including Malaria, tuberculosis, HIV and other sexually transmitted infections. Some of the questions will be of a personal nature. You are free to refuse to answer any questions if you don't want to. Whatever information you provide will be kept strictly confidential, and we won't include your name anywhere in the finished report.

At this time, do you want to ask me anything about the survey? Do you agree to participate in this survey?

YES………… NO………

GO TO THE NEXT HOUSE AND INFORM SUPERVISOR OF REFUSAL

THANK YOU

Signature of Interviewer:_________________________ Date:___________

VERIFY THE MOTHER HAS A CHILD OF 0-11 MONTHS OF AGE.
YOU CAN USE THE VACCINATION CARD OR MATERNAL CARD/BOOK TO VERIFY.

FOR ALL QUESTIONS IN THIS SURVEY, NEVER READ OUT THE PROVIDED OPTIONS/RESPONSES UNLESS THE INSTRUCTIONS STATE SO. WAIT FOR THE RESPONDENT TO ANSWER THE QUESTION AND THEN CIRCLE OR TICK THE APPROPRIATE OPTION/RESPONSE.

RESPONDENT'S BACKGROUND

NO.	QUESTIONS AND FILTERS	CODING CATEGORIES	GO TO
101	What is the name of your youngest Child<br>NAME_______________________		
102	What is the sex of [**NAME**]?	MALE………..…………….………..1<br>FEMALE………..……………………..2	
103	When was [**NAME**] born?<br>**IF CHILD WAS BORN MORE THAN 12 MONTHS AGO FIND ANOTHER HOUSE**<br>**IF MOTHER DOES NOT KNOW DATE ENTER 88**	DAY………………… [ ][ ]<br>MONTH……………. [ ][ ]<br>YEAR……..……… [ ][ ][ ][ ]	
104	**IF MOTHER DOES NOT KNOW (*NAME'S*) BIRTHDAY**<br>How old is (NAME?)	CHILD'S AGE IN COMPLETED MONTHS [ ][ ]	
105	When were you born?<br>**IF MOTHER DOES NOT KNOW DATE ENTER 88**	DAY………………… [ ][ ]<br>MONTH…………….. [ ][ ]<br>YEAR……..……….. [ ][ ][ ][ ]	
106	**IF MOTHER DOES NOT KNOW HER BIRTHDAY THEN ASK**<br>How old are you?	MOTHER'S AGE IN COMPLETED YEARS [ ][ ]	

NO.	QUESTIONS AND FILTERS	CODING CATEGORIES	GO TO
107	What is your current marital status?<br><br>**PROBE FOR SPECIFIC STATUS**	SINGLE, NO PARTNER..……...…………1<br>SINGLE, NON REGULAR PARTNER..…2<br>SINGLE WITH REGULAR PARTNER.…3<br>MARRIED……………………………...4<br>COHABITING……………………...…..5<br>WIDOWED……………………..……...6<br>DIVORCED/SEPARATED……………....7	
108	What is your highest level of education?	NEVER ATTENDED……..…………1<br>FUNCTIONAL ADULT LITERACY...…..2<br>INCOMPLETE PRIMARY…….………3<br>COMPLETE PRIMARY……………...4<br>O-LEVEL……..……….……………..5<br>A-LEVEL……………………………6<br>POST-SECONDARY...………............7<br>VOCATIONAL TRAINING....………8	

II. HCT/EMTCT

NO.	QUESTIONS AND FILTERS	CODING CATEGORIES	GO TO
**II a. HCT:**			
201a	Do you know the nearest place where you can be tested for HIV?	YES...………….........................1<br>NO…...……………………….…......2	→**202**
201b	**IF YES, RECORD NAME OF THE PLACE:___________________________**<br><br>**DISTANCE TO THE PLACE (KM)______________________________**<br>where one can be tested for HIV	LESS THAN 1 KM ……………...1<br>1 TO 5 KM …………...…………..2<br>OVER 5 KM (> 3 MILES)……….3	
202	Do you know any benefit of having an HIV test?	YES...…………………..… ..……1<br>NO….. ………………..………..2	→**204**

NO.	QUESTIONS AND FILTERS	CODING CATEGORIES	GO TO
203	What are the benefits of having an HIV test?<br><br>**CIRCLE AS MANY RESPONSES AS MENTIONED**	PLAN FUTURE ……….…….1<br>AVOID INFECTION …...........2<br>PROTECT UNBORN..……….3<br>GO FOR ART ………….........4<br>LEARN TO LIVE POSITIVELY ………………....5<br>FOOD SUPPORT……………..6<br>MATERIAL SUPPORT……….7<br>HIV CARE…………………….8<br>OTHER………….……..…..9<br>______<br>(**SPECIFY**)<br><br>DON'T KNOW………………88	
**II b. EMTCT:**			
204	Can the virus that causes AIDS be transmitted from a mother to her baby?	YES..…………………………...1<br>NO….. ………………………....2<br>DON'T KNOW……………….88	 ➔ 209<br>➔ 209
205	When can the virus that causes AIDS be transmitted from mother to her baby?<br><br>**CIRCLE AS MANY RESPONSES AS MENTIONED**	DURING PREGNANCY……...1<br>DURING DELIVERY……..….2<br>BREASTFEEDING………..… 3<br>OTHER…………………….….4<br>______<br>(**SPECIFY**)<br><br>DON'T KNOW ……………88	
206	Can the risk of a child getting the HIV infection from an infected mother be reduced?	YES..……………………..........1<br>NO….. ……………………..........2<br>DON'T KNOW……………..88	 ➔208a<br>➔208a

NO.	QUESTIONS AND FILTERS	CODING CATEGORIES	GO TO
207	What are the methods of reducing HIV infection from the mother to child<br><br>**CIRCLE ALL RESPONSES MENTIONED**	MOTHER DELIVERY IN THE HANDS OF A TRAINED HEALTH WORKER …...……………………1<br>USING ARVs……..…………..…..2<br>TESTING AND RECEIVING RESULTS FOR HIV…......………3<br>PREVENTION OF MALARIA DURING PREGNANCY….. …….4<br>BY OPERATING THE MOTHER (CAESERIAN SECTION)……………….................5<br>STI SCREENING, PREVENTION AND TREATMENT..……………..….....6<br>ATTENDING ANC. ……....……...7<br><br>**BABY**<br>USING ARV SYRUP …………..….8<br>SUPPLEMENTATION OF VITAMIN A & DEWORMING TABLETS.. ..……………….……..…9<br>REPLACEMENT FEEDING…...10<br>EXCLUSIVE BREAST FEEDING FOR FIRST SIX MONTHS…………………….….11<br>OTHER ....12<br>____________________<br>(**SPECIFY**)<br><br>DON'T KNOW ………………...88	
208a	Do you know a nearest place where you can get information and services related to reducing the risk of mother-to-child transmission of HIV?<br>____________________	YES..………………………………1<br>NO….. …………………………….2	➔**209**
208b	**IF YES, RECORD NAME OF THE PLACE:____________________**<br><br>**DISTANCE TO THE PLACE (KM)____________________**	LESS THAN 1 KM…………...…1<br>1 TO 5 KM ………..……………..2<br>OVER 5 KM (> 3 MILES)………3	
209	Did you go to a health facility for Antenatal Care when you were pregnant with (NAME)?	YES.…………………….………..1<br>NO…. ……………………….……...2	➔**219a**

NO.	QUESTIONS AND FILTERS	CODING CATEGORIES	GO TO
210	Can I please see your Maternal Card/Book for your pregnancy with NAME?<br><br>**INDICATE WHAT WAS PRESENTED CARD** ☐ or **BOOK** ☐	SHOWS CARD/BOOK ………….1<br>LOST CARD/BOOK ……………....2<br>NEVER HAD CARD/BOOK …....3<br>CARD/BOOK IN OTHER LOCATION ……………………..4<br>OTHER. …………………..……...5<br>________________<br>**(SPECIFY)**	 ➔**211b**<br>➔**211b**<br><br>➔**211b**<br>➔**211b**
211a	RECORD THE NUMBER OF ANC VISITS RECORDED ON THE MATERNAL CARD/BOOK DURING THE PREGNANCY WITH (NAME)<br>_______ / _______ / _______<br>DD MM YY<br>_______ / _______ / _______<br>DD MM YY<br>_______ / _______ / _______<br>DD MM YY<br>_______ / _______ / _______<br>DD MM YY<br>_______ / _______ / _______<br>DD MM YY	___________ # OF VISITS	➔**212**
211b	**CHECK QUESTION 209; IF THE MOTHER ATTENDED ANTENATAL BUT DOES NOT HAVE A CARD/BOOK, ASK IF SHE CAN REMEMBER THE NUMBER OF VISITS AND RECORD THEM**	___________ # OF VISITS	
212	During your pregnancy with [NAME] were you counseled about services that can reduce the risk of a mother transmitting the virus that causes AIDS to her child?	YES…...…….……………………1<br>NO….……………………………..2	
213	Were you counseled to take an HIV test during your pregnancy with [NAME]?	YES……..…….…………………..1<br>NO…….…………………………..…2	 ➔**219a**
214a	I do not want to know the results but when you were counseled did you take the HIV test?	YES……..………………………..1<br>NO……….. ………………………2	 ➔**218**

NO.	QUESTIONS AND FILTERS	CODING CATEGORIES	GO TO
214b	Were you tested for HIV with your husband/partner and given the test results together?	YES..……………………….……….1<br>NO.…. ……………………………2	
215a	You said you tested for HIV, where was the test done?<br><br>**PLACE**______________________________	HEALTH FACILITY..……………1<br>COMMUNITY..………………........2<br>OTHER …………………………..…3<br>________________________<br>**(SPECIFY)**	
215b	**DISTANCE TO THE PLACE IN KM WHERE THE HIV TEST WAS DONE**<br>**PLACE :** ____________________<br><br>**DISTANCE :** _________________	LESS THAN 1 KM ……………...…1<br>1 TO 5 KM …………..…………….2<br>OVER 5 KM (> 3 MILES).…………3	
216	I do not want you to tell me the results of the test, but did you find out the test result?	YES..…………………….………1<br>NO.…. ………………………...…2	➔**219a**
217	Why did you not find out the result?<br><br>**CIRCLE AS MANY RESPONSES AS MENTIONED**	HAD TO COME BACK FOR RESULT. ……………. …………..…1<br>AFRAID ………………….…...….... 2<br>ATTITUDE OF CLINIC STAFF........3<br>DID NOT WANT TO KNOW…........4<br>NO RESPONSE …………….........5<br>OTHER ………………………..........6<br>________________________<br>**(SPECIFY)**<br><br>DO NOT KNOW ……….……........88	
218	**ASK ONLY IF ANSWER FOR QUESTION 214a IS NO: RESPONDENT DID NOT TAKE HIV TEST, THOUGH COUNSELLED TO DO SO**<br><br>You said you did not take an HIV test, what could be the reasons for not taking a test?<br><br>**CIRCLE AS MANY RESPONSES AS MENTIONED**	LONG DISTANCE ...……..................1<br>NOT INTERESTED....………………..2<br>I DO NOT HAVE HIV……..................3<br>COST OF SERVICE……...…………..4<br>NOT NECESSARY…………………...5<br>AFRAID.………………..……….…6<br>TOO MUCH TIME……….................7<br>DON'T KNOW WHERE TO GO….....8<br>SERVICES NOT AVAILABLE..……9<br>STIGMA…………………………....10<br>OTHER ………………….................11<br>________________________<br>**(SPECIFY)**	

NO.	QUESTIONS AND FILTERS	CODING CATEGORIES	GO TO
219a	Where did you give birth to [NAME] ?	**HOME:**<br>MY HOME ……………………...…1<br>OTHER HOME …………………2<br><br>**HEALTH FACILITY:**<br>HOSPITAL ……………………...3<br>HEALTH CENTER (GOVT/<br>PNFP) ……………..…....………..4<br>CLINIC (PRIVATE)……..……...5<br>OTHER ……………..……………6<br>______________________<br>(**SPECIFY**)	 ➔**220**<br>➔**220**
219b	**IF RESPONSE IN QUESTION 219a IS HOSPITAL, HEALTH CENTER, OR CLINIC, RECORD NAME OF THE PLACE:**__________________________<br><br>**AND DISTANCE TO THE PLACE (KM)** _________________	LESS THAN 1 KM ……………....…1<br>1 TO 5 KM …………..……………..2<br>OVER 5 KM (> 3 MILES).…………3	
220	Who assisted you during the delivery of [NAME]	DOCTOR ….……………………1<br>CLINICAL OFFICER/<br>MEDICAL ASSISTANT…........2<br>NURSE/ MIDWIFE …………....3<br>NURSING AID...……….……..…4<br>TBA..……..…………..……...……...5<br>RELATIVE/FRIEND...…………6<br>NO ONE ……..……………...…7<br>OTHER …………………………..8<br>______________________<br>(**SPECIFY**)	

III. SEXUALLY TRANSMITTED INFECTIONS

NO.	QUESTIONS AND FILTERS	CODING CATEGORIES	GO TO
301	Have you ever heard of a sexually transmitted disease other than HIV/AIDS?	YES..……………………………...1<br>NO….. …………………..…………..2	→ 401
302	If a woman has a sexually transmitted disease, other than HIV/AIDS what signs/symptoms might she have?<br><br>**CIRCLE AS MANY RESPONSES AS MENTIONED**	LOWER ABDOMINAL PAIN………..1<br>ABNORMAL GENITAL DISCHARGE………..…….….......2<br>FOUL SMELLING DISCHARGE…...3<br>BURNING PAIN ON URINATION………………………..…4<br>BLOOD IN URINE…………………...5<br>SWELLING IN GENITAL AREA…...6<br>GENITAL SORES / HERPES……......7<br>GENITAL ITCHING………….....…....8<br>GENITAL WARTS……………..………..9<br><br>OTHER……..……...…..…………10<br>________________<br>(**SPECIFY**)<br><br>DON'T KNOW……….…….……….88	
303	If a man has a sexually transmitted disease, other than HIV/AIDS, what signs/symptoms might he have?<br><br>**CIRCLE AS MANY RESPONSES AS ARE MENTIONED**	LOWER ABDOMINAL PAIN….…….1<br>ABNORMAL GENITAL DISCHARGE……………………….....2<br>FOUL SMELLING DISCHARGE…....3<br>BURNING PAIN ON URINATION………………………..…4<br>BLOOD IN URINE………………….…..5<br>SWELLING IN GENITAL AREA…...6<br>GENITAL SORES/HERPES...…...….....7<br>GENITAL ITCHING…………….…....8<br>GENITAL WARTS...……….......…..9<br>OTHER..…………………………..…10<br>________________<br>(**SPECIFY**)<br><br>DON'T KNOW………….…...….88	

NO.	QUESTIONS AND FILTERS	CODING CATEGORIES	GO TO
304	What actions should one take when s/he has a sexually transmitted infection?<br><br>**CIRCLE AS MANY RESPONSES AS MENTIONED**	GO FOR TREATMENT…………....1<br>NOTIFY PARTNER(S)…………..2<br>COMPLETE TREATMENT………...3<br>USE CONDOM TILL CURED……4<br>ABSTAIN FROM SEX TILL CURED………………..……….…5<br><br>OTHER……………………...….6<br>______________________<br>(**SPECIFY**)<br><br>DON'T KNOW………………….88	
305a	Do you know where you could receive STI treatment if you had one?	YES..…………………..……….1<br>NO….. ………………...……….2	**➔401**
305b	**IF YES, RECORD NAME OF THE PLACE:**________________________<br><br>**DISTANCE TO THE PLACE (KM)**____________________________<br>**WHERE STI CAN BE TREATED**	LESS THAN 1 KM ……………..1<br>1 TO 5 KM …………..…………..2<br>OVER 5 KM (>3 MILES)………3	

IV. HIV KNOWLEDGE AND BEHAVIOURAL CHANGE

NO.	QUESTIONS AND FILTERS	CODING CATEGORIES	GO TO
401	Can you tell me the ways in which one can prevent the sexual transmission of HIV?<br><br>**CIRCLE AS MANY RESPONSES AS MENTIONED**	ABSTINENCE……………..……..1<br>BEING FAITHFUL………..…..2<br>CONDOM USE.………..………3<br><br>OTHER.…………….…………4<br>________________<br>**(SPECIFY)**<br><br>DON'T KNOW………..………88	
402	Can HIV be transmitted through the following ways?<br><br>**READ OPTION RESPONSES BELOW TO THE RESPONDENT**<br><br>1. Mosquito bites ………………………..……<br>2. Touching an infected person …………..<br>3. Sharing food with infected person …….<br>4. Sharing utensils with infected person….<br>5. Sharing toilets with infected person …..<br>6. Witchcraft ………………………………	YES NO DK<br><br>1 2 88<br>1 2 88<br>1 2 88<br>1 2 88<br>1 2 88<br>1 2 88	
403	Have you ever used either a male or female condom with a partner?	YES…..………………………...1<br>NO…….. ……………………....2	➔405

NO.	QUESTIONS AND FILTERS	CODING CATEGORIES	GO TO
404	If you have never used a condom, give reasons why?<br><br>**CIRCLE AS MANY RESPONSES AS MENTIONED**	DON'T KNOW WHERE TO GET IT FROM…………….…...1<br>NOT AVAILABLE IN MY AREA ……………………........2<br>CULTURAL / RELIGIOUS INHIBITIONS………………....3<br>PARTNER REFUSED……......4<br>BAD FOR MY HEALTH…......5<br>NOT NECESSARY ..……..…..6<br>COST…………………………..7<br>OTHER……………..…..……...8<br>________________________<br>(**SPECIFY**)	
405a	Where is the nearest place you can get condoms from?<br><br>**PLACE**___________________	SHOP..……………….……..…1<br>HOSPITAL...………………......2<br>HEALTH CENTRE.....………..3<br>PHARMACY/DRUG SHOP…..4<br>COMMUNITY DISTRIBUTER………………..5<br>FRIEND…………..…………..6<br>OTHER..…...…………………7<br>________________________<br>(**SPECIFY**)<br><br>DON'T KNOW……………..88	**➔501**
405b	**DISTANCE TO THE PLACE(IN KM)**<br>________________________<br>**WHERE YOU CAN GET CONDOMS FROM**	LESS THAN 1 KM ………………1<br>1 TO 5 KM …………...…………..2<br>OVER 5 KM (> 3 MILES)……….3	

IV. MALARIA

NO.	QUESTIONS AND FILTERS	CODING CATEGORIES	GO TO
501	When you were pregnant with (NAME), were you given any medicine to prevent malaria?<br><br>**PROBE TO FIND OUT IF SHE TOOK MALARIA MEDICATION WHILE NOT HAVING MALARIA**	YES..……………………..….1<br>NO……………………..…….2<br>DON'T KNOW ………..….88	 ➔505<br>➔505
502	Which medicine did you take during your pregnancy with (NAME) to prevent malaria?	SP/FANSIDAR……………......1<br>CHLOROQUINE…………..….2<br>CHLOROQUINE WITH FANSIDAR……………………3<br>QUININE……………………...4<br>HOMAPACK RED……………5<br>HOMAPACK GREEN………...6<br>COARTEM……………………7<br>SEPTRIN/COTRIMOXAZOLE.8<br>DAPZONE…………………….9<br>OTHER …………………… .10<br>______________________<br>(SPECIFY)<br><br>DON'T KNOW ……… ……. 88	 <br><br><br><br><br><br><br><br><br><br><br><br><br>➔505
503	How many times were you given the medicine to prevent malaria during pregnancy with [NAME]?<br><br>**PROBE TO FIND OUT HOW MANY TIMES MOTHER TOOK THE MEDICATION TO PREVENT MALARIA DURING PREGNANCY**	NUMBER OF TIMES ☐<br><br>OTHER …………………… .1<br>______________________<br>(SPECIFY)	
504	How many tablets were you given during your pregnancy with (NAME)?	☐☐<br># of TABLETS	

NO.	QUESTIONS AND FILTERS	CODING CATEGORIES	GO TO
505	How is malaria transmitted?	MOSQUITO BITES ....1<br>OTHER ………………….........2<br>________________<br>**(SPECIFY)**<br>DON'T KNOW 88	
506	How can you prevent malaria transmission?<br><br>**PROBE FOR MULTIPLE RESPONSES CIRCLE ALL MENTIONED**	SLEEPING UNDER TREATED MOSQUITO NETS …………......1<br>SLEEPING UNDER ANY MOSQUITO NET ....2<br>IN-DOOR HOUSE SPRAY ....3<br>PREVENTIVE TREATMENT...4<br>KEEPING HYGEINE IN COMPOUND ....5<br>OTHER ……………………......6<br>________________<br>**(SPECIFY)**<br>DON'T KNOW 88	
507	Do you have a mosquito net in this household?	YES..……………….……….....…1<br>NO.….. …………………….......2	→514
508	How many mosquito nets does your household have?	[ ][ ]<br># OF MOSQUITO NETS	
509	How long ago did your household obtain [NAME's] mosquito net?<br>***IF LESS THAN 1 MONTH, RECORD '00'.***	MONTHS AGO __________	
510	What type of mosquito net is [NAME's] bednet?<br><br>**PLEASE OBSERVE [NAME's] BEDNET.**	PERMANET…………………....1<br>SMARTNET...…………………2<br>OLYSET...……………………..3<br>KO-Net.………………………..4<br>SAFI-NET..……………………5<br>B52..…………………………...6<br>LUCKY NET..………………...7<br>PRETREATED NET.…………8<br>ORDINARY NET.……………9<br>OTHERS……………………...10<br>________________<br>**(SPECIFY)**	
511	When you got [NAME's] net, was it already treated with an insecticide to kill or repel mosquitoes?	YES..…………………….………1<br>NO…...………………………....2<br>DON'T KNOW ……………...88	
512	Since you got [NAME's] mosquito net, was it ever soaked or dipped in an insecticide to kill/repel mosquitoes or bugs?	YES..…………………….………1<br>NO…...………………………....2<br>DON'T KNOW ………….…...88	→514<br>→514

NO.	QUESTIONS AND FILTERS	CODING CATEGORIES	GO TO
513	How long ago was [NAME's] net last soaked or dipped in an insecticide to repel mosquitoes?<br><br>**IF LESS THAN 1 MONTH, RECORD '00'**	MONTHS AGO _______	
514	Did you sleep under a mosquito net when you were pregnant with [NAME]?	YES..……………….………..….1<br>NO…..………………………......2	 ➔**END**
515	How often did you sleep under a mosquito net when you were pregnant with [NAME]?	ALWAYS………………………1<br>SOMETIMES…………………..2<br>DO NOT REMEMBER………...3	

THANK YOU -THE END

Directional map to the Household
